# Brain Stimulation as a Therapeutic Tool in Amyotrophic Lateral Sclerosis: Current Status and Interaction With Mechanisms of Altered Cortical Excitability

**DOI:** 10.3389/fneur.2020.605335

**Published:** 2021-02-05

**Authors:** Federico Ranieri, Sara Mariotto, Raffaele Dubbioso, Vincenzo Di Lazzaro

**Affiliations:** ^1^Neurology Unit, Department of Neuroscience, Biomedicine and Movement Sciences, University of Verona, Verona, Italy; ^2^Department of Neurosciences, Reproductive Sciences and Odontostomatology, University of Naples “Federico II”, Naples, Italy; ^3^Unit of Neurology, Neurophysiology, Neurobiology, Department of Medicine, Campus Bio-Medico University, Rome, Italy

**Keywords:** cortical hyperexcitability, repetitive transcranial magnetic stimulation, transcranial direct current stimulation, neuromodulation, functional status, survival, non-invasive brain stimulation, ALS

## Abstract

In the last 20 years, several modalities of neuromodulation, mainly based on non-invasive brain stimulation (NIBS) techniques, have been tested as a non-pharmacological therapeutic approach to slow disease progression in amyotrophic lateral sclerosis (ALS). In both sporadic and familial ALS cases, neurophysiological studies point to motor cortical hyperexcitability as a possible priming factor in neurodegeneration, likely related to dysfunction of both excitatory and inhibitory mechanisms. A trans-synaptic anterograde mechanism of excitotoxicity is thus postulated, causing upper and lower motor neuron degeneration. Specifically, motor neuron hyperexcitability and hyperactivity are attributed to intrinsic cell abnormalities related to altered ion homeostasis and to impaired glutamate and gamma aminobutyric acid gamma-aminobutyric acid (GABA) signaling. Several neuropathological mechanisms support excitatory and synaptic dysfunction in ALS; additionally, hyperexcitability seems to drive DNA-binding protein 43-kDA (TDP-43) pathology, through the upregulation of unusual isoforms directly contributing to ASL pathophysiology. Corticospinal excitability can be suppressed or enhanced using NIBS techniques, namely, repetitive transcranial magnetic stimulation (rTMS) and transcranial direct current stimulation (tDCS), as well as invasive brain and spinal stimulation. Experimental evidence supports the hypothesis that the after-effects of NIBS are mediated by long-term potentiation (LTP)-/long-term depression (LTD)-like mechanisms of modulation of synaptic activity, with different biological and physiological mechanisms underlying the effects of tDCS and rTMS and, possibly, of different rTMS protocols. This potential has led to several small trials testing different stimulation interventions to antagonize excitotoxicity in ALS. Overall, these studies suggest a possible efficacy of neuromodulation in determining a slight reduction of disease progression, related to the type, duration, and frequency of treatment, but current evidence remains preliminary. Main limitations are the small number and heterogeneity of recruited patients, the limited “dosage” of brain stimulation that can be delivered in the hospital setting, the lack of a sufficient knowledge on the excitatory and inhibitory mechanisms targeted by specific stimulation interventions, and the persistent uncertainty on the key pathophysiological processes leading to motor neuron loss. The present review article provides an update on the state of the art of neuromodulation in ALS and a critical appraisal of the rationale for the application/optimization of brain stimulation interventions, in the light of their interaction with ALS pathophysiological mechanisms.

## Introduction

Amyotrophic lateral sclerosis (ALS) is a fatal neurodegenerative disease affecting the cortical, brainstem, and spinal motor neurons, leading to death due to respiratory failure usually within 3–5 years of symptom onset. While about 90% of cases are sporadic, the remaining are familial and usually inherited as dominant traits. Both familial and sporadic cases have been associated with mutations of several genes regulating protein homeostasis, RNA homeostasis, and cytoskeletal dynamics, with common downstream neuropathological and phenotypical alterations (1).

Many studies, investigating neurophysiological and neuropathological alterations in both familial and sporadic ALS, converge on a “dying-forward” hypothesis at the origin of the disease, with cortical hyperexcitability as a possible driver of neurodegeneration. In this context, different molecular alterations of glutamatergic and gamma aminobutyric acid (GABA)-ergic neurons and receptors and of astrocytes, as well as the excitation/inhibition imbalance of motor cortical circuits, might all contribute the final picture of corticospinal hyperexcitability. These alterations suggest the possibility of exploiting therapeutic strategies pointing to hyperexcitability, targeted to specific molecular and/or functional alterations. Such a therapeutic approach is further valued by recent evidence that early cortical hyperexcitability is sufficient to trigger molecular alterations commonly associated with ALS (2), possibly initiating upper (UMN) and lower motor neuron (LMN) damage.

Current pharmacological therapeutic attempts point to excitotoxicity control and neuroprotection. To date, the only two drugs approved for the treatment of ALS, riluzole, and edaravone, are targeted to mechanisms of excessive excitability of corticospinal neurons (CSNs). Both drugs show modest effects on disease progression (3). Riluzole, available from 1995, acts by reducing glutamatergic transmission, through pre-synaptic inhibition of glutamate release or post-synaptic modulation of ionotropic glutamate receptors, in addition to inactivation of voltage-gated Na^+^ channels, and it was shown to determine an increase of ~3 months in median survival time (4–6). Edaravone, approved by the Food and Drug Administration in 2017, is thought to act as a free radical scavenger preventing oxidative damage and showed a slight slowing in disease progression at 6 months in a selected group of early affected patients without respiratory failure (7). However, this effect seems to vanish at a longer follow-up (8).

Starting from 2004, different non-invasive brain stimulation (NIBS) techniques have been employed to modulate motor cortical excitability in the attempt to counteract excitotoxicity. The main attempts have been made with repetitive transcranial magnetic stimulation (rTMS), allowing repeated activation of cortical synapses, and with transcranial direct current stimulation (tDCS), acting on resting membrane potential: both techniques can produce a prolonged suppression of neuronal excitability through modulation of synaptic plasticity in physiological conditions (9). Additionally, invasive stimulation of the motor cortex and of the corticospinal tract by means of epidural electrodes has been attempted, with the main purpose of performing a chronic and possibly more effective stimulation. Since these approaches of brain stimulation exert their modulatory effects by acting at different levels of intracortical circuitry, they could be used to target specific pathogenic mechanisms.

In this review, we provide an update on brain stimulation interventions applied in ALS and on their potential benefits and current limitations, in the light of their interaction with mechanisms of corticospinal excitability.

## Dysfunction of Corticospinal Circuits

A full knowledge of ALS pathogenesis is still to be uncovered; however, the known pathogenic mechanisms and resulting dysfunctions are fundamental in developing targeted therapeutic interventions.

The two main histological characteristics of the disease, described by the terms “amyotrophic” and “lateral sclerosis,” derive from the degeneration of motor neurons in the anterior horn of the spinal cord and from axonal loss in the lateral columns (the lateral corticospinal tract), respectively.

After the initial Charcot's hypothesis on the primacy of corticomotoneuronal dysfunction (10), the so-called “dying-forward” hypothesis, two alternative explanations have been considered, based on either a dying-back mechanism secondary to LMN dysfunction (11) or an independent process of upper and lower motor neuron degeneration (12).

In the last three decades, accumulating evidence supports the initial hypothesis of the hyperexcitability of the corticomotoneuronal system as a leading pathogenic mechanism. This view is supported by several elements: (1) prominent muscle wasting in districts with direct corticospinal projections and sparing of LMN not controlled by corticospinal tract (i.e., oculomotor and Onuf's nuclei motor neurons); (2) time-dependent spreading of neuropathological alterations from layer V pyramidal cells to structures under direct control of corticofugal projections; (3) clinical syndromes characterized by frontal dysfunction preceding muscle weakness; (4) neurophysiological evidence of hyperexcitable corticospinal circuits; (5) cortical hyperexcitability documented before LMN dysfunction; and (6) evidence of altered glutamatergic signaling [reviewed in Eisen et al. (13)].

Based on the above considerations, it is assumed that hyperexcitability represents a common pathogenic mechanism leading to excitotoxicity and degeneration of upper and lower motor neurons. Hyperexcitability can be mediated either by direct pre-synaptic mechanisms (i.e., overstimulation), due to increased glutamate stimulation, or by indirect post-synaptic mechanisms (i.e., overreaction), due to altered interneuron regulation, glutamate receptors, or intrinsic motoneuronal excitability (14).

The link between UMN hyperexcitability and LMN degeneration remains a matter of debate, and a mechanism of glutamate-mediated excitotoxicity from the direct corticospinal connection is not demonstrated. Indeed, findings on alterations of spinal circuit excitability in ALS are not conclusive, as different pathways and interneurons characterize animal models and humans. We refer the reader to existing literature for details on the complexity of spinal circuit pathophysiology [e.g., Gunes et al. (15) for a recent review].

### Neurophysiological Insights

The introduction of transcranial magnetic stimulation (TMS) allowed to activate non-invasively the corticospinal motor pathways, both directly and trans-synaptically, and then to study the function of corticospinal projections and intracortical circuits in physiological and pathological conditions (16). In ALS, specific TMS protocols allowed to demonstrate an altered excitability of CSNs and of cortical inhibitory interneurons connected with CSNs [reviewed in Vucic et al. (17)].

The basic output of TMS of the primary motor cortex (M1), the motor evoked potential (MEP) recorded from a target muscle, is the result of activation of the different levels of the motor pathway, and hence, at central level, it is influenced by the excitability of upper and lower motor neurons, of cortical and spinal/brainstem interneurons, as well as by the motor neuron pool (i.e., the integrity of motor pathway). Among specific single-pulse TMS parameters of corticospinal excitability, motor threshold (MT) is defined as the minimum TMS intensity required to elicit the MEP. Intensity–response curve of MEP amplitude reflects recruitment of the CSN pool at increasing stimulation intensities. Additional protocols allow to study inhibitory intracortical phenomena. The silent period of electromyographic activity, induced by a single suprathreshold TMS pulse of the contralateral M1, partly depends on the activation of cortical GABA-B receptors and can be used as an index of activation of cortical inhibitory interneurons (18). Additionally, a paired-pulse TMS protocol, in which a subthreshold pulse precedes of 1–5 ms a suprathreshold test pulse, produces a “short-interval” intracortical inhibition (SICI) (19) of the test response, which is believed to be mediated by the activation of cortical GABA-A receptors (20, 21).

Abnormalities of the above parameters in both sporadic and familial forms of ALS (i.e., reduced MT, increased MEP amplitude and recruitment, shorter/absent silent period, and reduced SICI) point to the occurrence of phenomena of concomitant increased CSN excitability and of reduced intracortical inhibitory activity (17). Interestingly, the above alterations have been observed in the early and pre-symptomatic stages of sporadic and familial cases of ALS, respectively, supporting the hypothesis that hyperexcitability can precede neurodegeneration (22–25). However, it is not clear how TMS metrics change over time, as conflicting results exist in literature. The first longitudinal study (26) failed to find a clear change of TMS parameters over time. Subsequent studies have reported an initial reduction of MT, followed by a progressive and eventual cortical inexcitability in later stages (22, 27–29). A recent prospective study on a large cohort of ALS patients (*n* = 345), with a mild–moderate disease severity [mean ALS functional rating scale (ALSFRS-R): 40.5] at the time of neurophysiological investigation, reports that cortical hyperexcitability increases with disease duration, excluding subjects with inexcitable M1 to TMS (30). Of note, in this study, a marked reduction of excitability or inexcitability to TMS was found in 21% of ALS patients: the occurrence of inexcitability is expected to be higher in later stages due to progressive motor neuron depletion (31, 32).

It remains an open question whether hyperexcitability represents, at least in part, a compensatory phenomenon of motor neuron degeneration (33). While increased excitability following reduced input is a known physiological phenomenon (34), this hypothesis in ALS is not confirmed by the fact that ALS mimics (i.e., other neuromuscular disorders, such as motor neuropathies, spinal muscular atrophy, Kennedy's and Hirayama's diseases, hereditary spastic paraplegia, neuromyotonia, and myopathies) do not exhibit cortical hyperexcitability (30). However, it cannot be excluded that genetically altered motor neurons (35) are more sensitive to the loss of cortical inhibitory inputs.

Growing body of evidence also reveals that ALS exhibits subclinical peripheral and central sensory neuron dysfunctions (36–39). Indeed, somatosensory evoked potential (SEP) studies have demonstrated an hyperexcitability of somatosensory cortex, as indicated by enlarged early component of cortical SEP (i.e., N20, P25), which was more evident in moderate respect to severe cases of ALS (40) and was associated to shorter survival (38). In addition, a paired-pulse SEP study demonstrated a marked reduction of the physiological inhibition, putting forward an impaired inhibition as a mechanism underlying cortical hyperexcitability (39) and closely resembling the TMS data obtained for M1. However, if this sensory cortical hyperexcitability could reflect a multisystem neurodegenerative disorder (41) or represent a compensatory upregulation mechanism related to the functional impairment of the motor cortex is still debated.

Despite the entire picture of pathophysiological mechanisms of hyperexcitability in ALS remains to be uncovered, the known physiological alterations support the rationale of interventions based on reducing corticospinal activity aimed at preventing upper and/or lower motor neuron excitotoxicity.

### Neuropathological Alterations

Although specific mechanisms underlying selective degeneration of motor neurons in ALS remain unknown, some neuropathological alterations might underlie degeneration, abnormal cell excitability, and synaptic transmission and thus account for cortical circuit dysfunction. Conversely, neuronal hyperactivity can lead to molecular alterations accounting for cell dysfunction and degeneration.

#### Key Aspects of Neuronal Degeneration

Genetic studies have identified a high number of genes associated with both familial and sporadic ALS; however, both forms show a substantial convergence in their pathological features leading to motor neuron degeneration (42).

The neuropathological hallmark of the disease is the presence of transactivation response DNA-binding protein 43-kDA (TDP-43) positive inclusions into neuronal cytoplasm (43, 44) frequently as skein-like inclusions. Small intracellular eosinophilic inclusions (the so-called Bunina bodies) are also observed in the spinal cord and brainstem nuclei and are considered highly specific for the disease (45). Microscopic examination shows loss of myelinated axons and degeneration of neurons in the lateral/anterior columns of the spinal cord, brainstem motor nuclei, and motor cortex, associated with vacuolization, spongiosis, and reactive astrogliosis (46). The activation of microglia, which occasionally shows TDP-43 deposits, further causes the release of proinflammatory molecules thus supporting inflammation (46). These aspects are observed in all cases of ALS, which clinical variability seems to depend on the location of the neuropathological changes, and in particular on the neuroanatomical distribution of TDP-43 pathology, rather than to the presence of specific pathological aspects. UMNs and LMNs together with the frontotemporal cortex and subcortical regions are most vulnerable to ALS pathology. However, neurodegeneration progresses to different areas including the amygdala, substantia nigra, and striatum (47), and it is potentially explained by the cell-to-cell spread of the disease, similar to what is seen in prion disease (48, 49).

The exact mechanisms of TDP-43-induced cell death are currently unclear. Reduced TDP-43 seems to induce defect in cell proliferation rather than directly cause cell death. However, overexpression of wild-type and mutant TDP-43 can induce p53-dependent apoptosis of neuronal progenitors and TDP-43 *per se* can promote the expression of proapoptotic genes. In mouse models, cytoplasmic mislocalization of TDP-43 induces apoptosis even in the absence of aggregates. In addition, aberrant TDP-43 alters splicing and RNA metabolism. Finally, aggregates of hyperphosphorylated and fragmented TDP-43 protein are associated with stress responses and facilitate cell death (50). In particular, TDP-43 has a fundamental role in regulating the survival of motor/cortical neurons and astrocytes through its effects on stress granules, which facilitate cell survival in course of exposure to stress (51). These aspects are the basis of the significant cortical degeneration/dysfunction observed in ALS. In particular, the prominent TDP-43 accumulation in cortical neurons is directly connected with cortical dysfunction. The most vulnerable structures are: the pyramidal cells in layer Vb of M1; large alpha motor neurons of the lower brainstem and spinal cord; projection neurons of the striatum; parvocellular red nucleus; inferior olive; and motor nuclei of the V, VII, XI, and XII cranial nerves. The subsequent diffusion through corticofugal projections underlines the primary involvement of the cerebral cortex (13).

#### Link to Hyperexcitability

Hyperexcitability substantially originates from reduced threshold to respond to a stimulus, or exaggerated response to a suprathreshold stimulus, being it referred to individual cells or entire cortical networks.

Investigations on gene expression in the cortex from ALS patients or mouse models revealed a wide series of transcriptomic alterations involving channels, transporters, and receptors. Specifically, these abnormalities concern Na^+^/K^+^ ATPase, voltage-gated Na^+^, and K^+^ channels, K^+^/Cl^−^ co-transporter, ionotropic and metabotropic glutamate receptors, glutamate transporters, and GABA receptors [reviewed in Brunet et al. (52)]. Such alterations account for dysfunction of individual cell excitability and of excitatory and inhibitory synaptic transmission.

Excessive response to stimuli of cortical cells is sustained by ion channel dysfunctions. In particular, persistent Na^+^ currents together with reduction of K^+^ conductance lead to an imbalance of ion channels, which finally increases membrane excitability (35). Another crucial aspect of cortical hyperexcitability is the reduced glutamate clearance by astrocytes, which seems to cause motor neuron overstimulation and consequently neuronal degeneration, possibly through increased Ca^2+^ concentration (35). Indeed, increased Ca^2+^ flux through glutamate receptors and activation of voltage-gated channels are crucial in determining excitability of motor neurons, which are particularly sensitive to Ca^2+^ levels (53). Astrocytes might also favor cortical hyperexcitability through dysregulation of potassium clearance, which leads to extracellular K^+^ accumulation and consequently membrane depolarization and increased excitability (35). In cultured cortical neurons, increased extracellular Na^+^, Ca2^+^, K^+^, and Cl^−^ levels lead to hyperexcitability and increase of dendritic spines, thus supporting the role of these changes in inducing early toxicity (54).

A major role in UMN overstimulation is currently attributed to the impairment of cortical inhibitory circuits due to the involvement of GABAergic interneurons, which alters GABA receptor-mediated inhibitory currents and facilitates motor neuron degeneration (35, 55). Pre- and post-synaptic glycine receptors, which mediate synaptic inhibition, are also involved in motor neuron/interneuron dysfunction (56). GABAergic interneurons account for 20–25% of all neocortical neurons and can be distinguished, based on non-overlapping molecular markers, in three main groups that also show specific synaptic connections to layer V pyramidal cells: (1) parvalbumine (PV)-positive neurons, representing the largest interneuron population in the neocortex, include two subgroups projecting, respectively, to axon initial segment and to soma or proximal dendrites of pyramidal cells; (2) somatostatine (SST)-positive neurons receive strongly facilitatory inputs and project to apical dendrites of pyramidal cells; and (3) 5HT_3A_R-positive neurons, further divided into vasointestinal peptide (VIP)-positive neurons, providing inhibitory input to SST+ cells, and VIP-negative neurons, connected to the soma or dendrites of pyramidal cells through GABAA or GABAB receptors (57). These cell populations can be affected in ALS in terms of cell loss (58, 59) or altered excitability (60). Interestingly, in a TDP-43 mouse model, it has been observed that hyperactivity of SST+ interneurons can be responsible for hyperexcitability of layer V pyramidal cells through inhibition of PV+ interneurons (60). Moreover, a demonstration of degeneration of PV+ interneurons along with reduced expression of GABAA receptor alpha-1 subunit in ALS patients can account for reduced intracortical inhibition (i.e., SICI), as tested by TMS (30). Moreover, increasing the activity of hypoactive PV+ interneurons in a SOD1-G93A mouse model was recently demonstrated to reduce CSN hyperexcitability and to delay the onset of motor deficits (61).

At a circuit level, the activity of CSNs and interneurons is regulated by several afferent projections from distant structures by means of different neurotransmitters. Neuropathological studies on both ALS patients and mouse models have shown impairment of serotoninergic, dopaminergic, noradrenergic, histaminergic, and cholinergic systems, variably characterized by loss of neurons, altered expression of transporters and receptors, or TDP-43 inclusions [reviewed in Brunet et al. (52)]. However, it is not defined to which extent each of these alterations of cortical afferents might contribute to motor cortical hyperexcitability.

Altogether, the above dysfunctions contribute to overactivation of CSNs, which can subsequently induce the degeneration of spinal motor neurons. In addition, it has been recently demonstrated that neuronal hyperactivity leads to the upregulation and accumulation of a highly insoluble isoform of TDP-43 within neurons and glia, supporting the hypothesis of an upstream pathogenic role of hyperexcitability in ALS (2).

#### Synaptic Dysfunction

As in other neurodegenerative conditions, synapse degeneration is a crucial aspect of ALS. In mouse models, mutated TDP-43 pathological accumulation alters dendritic spine density and causes synaptic alterations leading to excitability dysfunction and finally, neuronal damage (62).

In human post-mortem tissues, high sensitive analyses allowed to demonstrate that synapse loss occurs also in the absence of neuron loss. Despite the exact cause of synapse loss is unknown, it could be due to accumulation of TDP-43 aggregates into spines and/or to direct phagocytosis mediated by activated microglial cells (63). The alteration of dendrite and synapse formation/morphology leads to accumulation of glutamate with subsequent overactivation of glutamate receptors, which finally promotes the cell death cascade. In support of the relevance of these pathological changes, recent therapeutic approaches besides riluzole, proposed for clinical trials, target synaptic alterations and, specifically, receptors, ion channels, and hyperexcitability (64).

## Targets of Brain Stimulation Techniques

Different non-invasive brain stimulation methods have been evaluated in ALS with the purpose of producing persistent modulatory effects on neural activity by interfering with mechanisms of cortical motor neuron hyperexcitability. Invasive epidural cortical stimulation was also tested for chronic administration. While these interventions share the effect of an overall reduction of the corticospinal output to subsequent depolarizing inputs, which might prevent excitotoxicity, we have now sufficient knowledge on specific underlying mechanisms characterizing different types and paradigms of brain stimulation. Focusing on the interaction of brain stimulation with specific mechanisms characterizing hyperexcitability in ALS might represent the proper way toward targeted neuromodulatory interventions with increased therapeutic potential.

Among techniques of non-invasive brain stimulation, rTMS produces long-lasting effects on synaptic transmission by repeated synaptic activation following depolarizing inputs (65), and inhibitory (LTD-like) effects can be obtained with either low-frequency (e.g., 1 Hz) stimulation (66), patterned paradigms such as the continuous theta burst stimulation (cTBS) (67), or paired associative stimulation protocols (68). Instead, tDCS acts by producing membrane depolarization/hyperpolarization that is subthreshold for evoking action potentials but that can influence the neuron spontaneous firing frequency (69) and long-term potentiation (LTP)/long-term depression (LTD) mechanisms of synaptic plasticity (70–72).

Recordings from the corticospinal tract in intact human subjects through implanted epidural electrodes provided insight into the site of action of different protocols of brain stimulation (73). Indeed, the corticospinal discharge represents a direct measure of the M1 output being not influenced by spinal excitability. It is then possible to test the effect of neuromodulatory interventions on the corticospinal activity generated by single TMS pulses with known patterns of activation of corticospinal circuits. Specifically, depending on how M1 is activated with single-pulse TMS, the descending corticospinal volley can be composed of the following: (1) a very early wave generated by the direct activation of corticospinal axons, hence termed “D wave;” (2) an indirect “I1 wave” generated by monosynaptic activation of CSNs; and (3) one or more later I waves generated by the transsynaptic activation of CSNs at a greater distance from the cell body or by reverberating activity of a local intracortical circuit [reviewed in Di Lazzaro et al. (16)].

Corticospinal recordings, as a first point, confirm the cortical origin of LTP-/LTD-like effects produced by brain stimulation, since they demonstrate changes in the composition and amplitude of the descending corticospinal volleys generated by the activation of cortical circuits. Additionally, effects on individual components of the corticospinal volley, generated by direct activation of CSNs or by the indirect activation of CSNs through corticocortical projections, point to specific sites of action of rTMS and tDCS protocols [reviewed in Di Lazzaro and Rothwell (73)].

Most NIBS protocols selectively modulate the later I waves, indicating a modulatory effect either on interneurons connected to CSNs (74) or on a reverberating local circuit within M1, composed of interconnected layer II/III excitatory intracortical pyramidal neurons, layer V pyramidal tract neurons, and GABAergic interneurons (75).

This is the case of most inhibitory paradigms, such as 1-Hz rTMS (76), paired associative stimulation (PAS) at an interstimulus interval (ISI) of 10 ms (77), and cathodal tDCS (78). Some facilitatory paradigms, such as intermittent TBS (iTBS) and PAS at an ISI of 25 ms, show the same pattern of cortical interaction though in an opposite direction (79, 80). Differently, the inhibitory cTBS protocol selectively reduces the I1 wave: this indicates that it affects the excitability of the initial monosynaptic input to CSNs (81).

Interestingly, while an effect on later I waves has been demonstrated for both cathodal (inhibitory) and anodal (facilitatory) tDCS, anodal tDCS also showed facilitation of the I1 wave (78) and of the D wave (82); the latter finding indicates increased excitability of corticospinal axons and this was not tested with cathodal tDCS. Since the effects of tDCS on cortical plasticity are polarity-specific and are likely to be primed by depolarizing/hyperpolarizing effects on neuronal membrane (83), it can be hypothesized that cathodal tDCS also has a direct modulatory effect on corticospinal axons in analogy with anodal tDCS but in the opposite direction.

Moreover, a comparison of descending volleys evoked by transcranial and epidural motor cortex stimulation indicates that epidural stimulation can produce repetitive excitation of CSNs similar to TMS (84), supporting the use of epidural stimulation to modulate cortical excitability in the same way as rTMS protocols, even if the effect of prolonged stimulation on corticospinal descending activity was not investigated.

**Figure 1:**
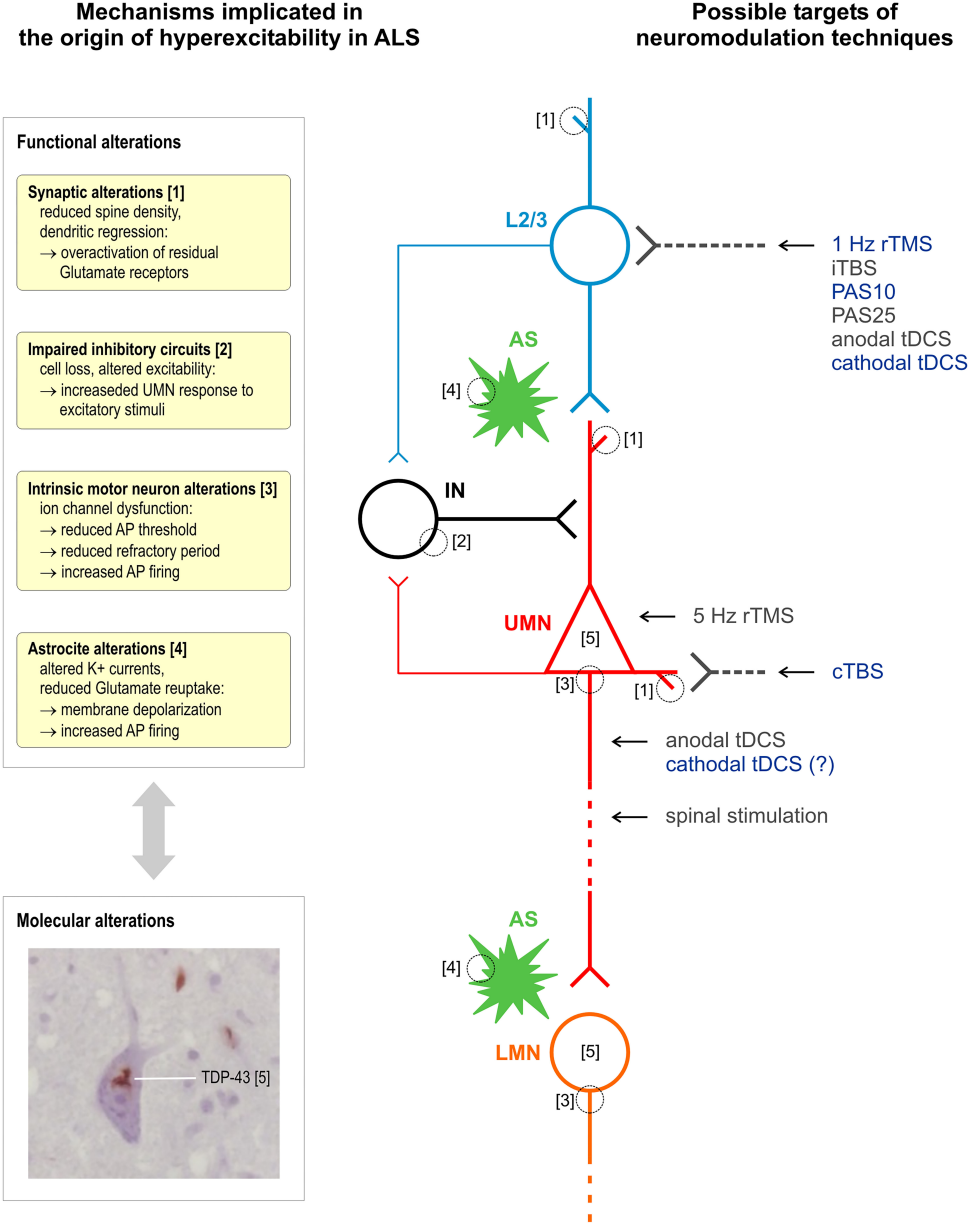
Schematic representation of mechanisms implicated in the origin of hyperexcitability in ALS and of proposed sites of interaction of different techniques of stimulation of the corticospinal system. Excitatory glutamatergic input to upper motor neurons (UMN, red) is mainly provided by upstream layer 2 and 3 pyramidal neurons (L2/3, light blue) and modulated by astrocytes (AS, green). Different populations of interneurons (IN, black) provide GABAergic input within M1 (a single simplified connection to UMN apical dendrite is represented). All the above cell groups and synaptic connections can be affected by ALS pathophysiological alterations, and current evidence suggests an interplay between functional and molecular alterations, such as TDP-43 cytoplasmic accumulation. It is proposed that most NIBS protocols selectively modulate bursting cells of layers 2 and 3 that project upon layer 5 pyramidal cells or a reverberating local circuit within M1 including GABAergic interneurons; cTBS selectively suppresses the excitability of monosynaptic connections to CSNs; 5-Hz rTMS may produce its effects by enhancing the excitability of CSNs; anodal tDCS and other invasive and non-invasive spinal stimulation techniques interact with corticospinal axons. Stimulation protocols with a documented inhibitory effect on corticospinal excitability are indicated in blue. Histological insert shows TDP-43 skein-like inclusions in the cytoplasm of motor neurons of the hypoglossal nuclei in the medulla oblongata (×100 original magnification). Neuromodulation techniques—rTMS, repetitive transcranial magnetic stimulation; tDCS, transcranial direct current stimulation; iTBS/cTBS, intermittent/continuous theta burst stimulation; PAS, paired associative stimulation.

Figure 1 summarizes the proposed sites of interaction of different stimulation techniques within intracortical and corticospinal circuits affected by ALS.

It must be considered that the effects of modulatory interventions on corticospinal activity were measured in a small number of subjects: this limits the level of certainty of proposed models of interaction of different brain stimulation protocols with motor cortical circuits. However, based on current evidence, we can expect that certain types of interventions suppressing M1 excitability, such as cTBS, will mainly affect excitatory intracortical inputs to layer V pyramidal cells, while other protocols, such as 1-Hz rTMS, PAS, and cathodal tDCS, will affect more complex intracortical circuits including GABAergic interneurons connected to layer V pyramidal cells. Moreover, based on the biophysical properties of the technique and on very preliminary data, it is conceivable that tDCS has also some direct modulatory effect on CSN excitability in addition to its interaction with synaptic plasticity (85, 86).

## Therapeutic Trials in ALS

A systematic search for original studies applying brain or spinal stimulation on ALS subjects, with the purpose of persistent neuromodulation aimed at affecting functional status, was performed on 10th August 2020 in the PubMed database (U.S. National Library of Medicine, NIH), using the following search terms:

#1 [als amyotrophic lateral sclerosis (MeSH Terms)] OR [motor neuron disease (MeSH Terms)] OR [amyotrophic lateral sclerosis (Title/Abstract)] OR [ALS (Title/Abstract)];
#2 [transcranial magnetic stimulation (MeSH Terms)];
#3 [electrical stimulation of the brain (MeSH Terms)];
#4 [magnetic field therapy (MeSH Terms)];
#5 [stimulation (Title/Abstract)];
#6 {[magnetic (Title/Abstract)] AND [repetitive (Title/Abstract)]} OR [current (Title/Abstract)] OR [electrical (Title/Abstract)] OR [epidural (Title/Abstract)] OR [spinal (Title/Abstract)] OR [focused ultrasound (Title/Abstract)] OR [static magnetic field (Title/Abstract)];
#7 [TMS (Title/Abstract)] OR [tDCS (Title/Abstract)] OR [tACS (Title/Abstract)]
Combined query: #1 AND [#2 OR #3 OR #4 OR (#5 AND #6) OR #7].

Query produced 431 results, which were screened on the basis of title and abstract; 28 remaining full-text articles were assessed for eligibility; 14 original studies were included in qualitative synthesis (summarized in Table 1).

**Table 1:** Summary of studies applying brain or spinal stimulation with the purpose of producing persistent effects on functional status in ALS.

	**Design**	**Protocol**	**Site**	**Dosage**	**Duration**	***N***	**Control**	**Riluzole**	**Outcome**	**Main findings**
**rTMS studies**										
Di Lazzaro et al. (87)	Pilot	1 Hz	M1 (L + R)	10 d/4 m	25, 30 m	2	No	No	Norris, MRC	Slowed progression (Norris, MRC)
Di Lazzaro et al. (87)	Pilot	20 Hz	M1 (L + R)	8 d/m	2, 3 m	2	No	Yes	Norris, MRC	No detectable effects
Di Lazzaro et al. (88)	RCT	cTBS	M1 (L + R)	5 d/m	6 m	20	Yes	Yes	ALSFRS-R, MRC	Slowed progression (ALSFRS-R)
Zanette et al. (89)	RCT	5 Hz	M1 (L + R)	5 d/w	2 w	10	Yes	Yes	ALSFRS-R, MRC, MVIC (grip), FSS (fatigue), SF-36 (QoL)	Improved grip strength and QoL at the end of treatment, not significant at 2 weeks after the end of treatment
Di Lazzaro et al. (90)	RCT	cTBS	M1 (L + R)	5 d/m	12 m	20	Yes	Yes	ALSFRS-R, MRC	No significant effects
Di Lazzaro et al. (91)	Pilot	cTBS	M1 (L + R)	5 d/m	26 m	1	No	Yes	ALSFRS-R	Slowed progression
Di Lazzaro et al. (92)	Open label	cTBS	M1 (L + R)	5 d/m	5, 10, 12 m	3	No	Yes	ALSFRS-R, Resp. failure	Slowed progression compared with previous 1-year period of observation
Di Lazzaro et al. (93)	Pilot	cTBS	M1 (L + R)	10 d/m	12 m	5	No	n/a	ALSFRS-R	No significant effects vs baseline progression. Slower progression than control patients and possibly slower progression than patients treated 5 d/m over 6 months in a pooled analysis with data from Di Lazzaro et al. (88) and Di Lazzaro et al. (90)
**tDCS studies**										
Di Lazzaro et al. (94)	Pilot	c-tDCS	M1 (L + R)	1 mA, 20 min; 5 d/m	1, 12 m	2	No	n/a	ALSFRS-R, Resp. failure	No detectable effects (1 patient died after 1 month of treatment)
Benussi et al. (95)	RCT	tDCS	M1 (a), cervical spine (c)	2/4 mA, 20 min; 5 d/w	2 w	30	Yes	Yes	ALSFRS-R, MRC, ALSAQ-40, EQ-5D, EQ-VAS, CBI	Improvement/stabilization in MRC, EQ-VAS, CBI clinical scores
Madhavan et al. (96)	Pilot	a-c-sham-tDCS	M1 (L)	2 mA, 20 min; 3 d/w	4 w	1	No	No	ALSFRS-R, 6MWD, 10MWT, TUG	No detectable effects
Sivaramakrishnan et al. (97)	Pilot	a-tDCS	M1 (R)	2 mA, 20 min; 3 d/w	8 w	2	No	No	ALSFRS-R, 6MWD, 10MWT, TUG, FSS, BDI	No detectable effects
**Invasive stimulation studies**										
Sidoti and Agrillo (98)	Pilot	Subdural ES (30 Hz)	M1 (L + R)	Continuous	2 y	4	No	n/a	ALSFRS	Slowed progression in 2 out of 4 patients
Di Lazzaro et al. (91), Di Lazzaro et al. (99)	Pilot	Epidural ES (3 Hz; 30 Hz)	M1 (L + R)	Continuous	>3 y	1	No	n/a	ALSFRS-R	No effect on disease progression in the first 2 years of treatment. Survival at 12 years after prolonged epidural ES treatment
Di Lazzaro et al. (100)	Pilot	Epidural ES (60 Hz; burst)	Cervical spine	Continuous	10 m	1	No	Yes	ALSFRS-R	No detectable effects

*Study design—RCT, randomized controlled trial. Stimulation protocols—cTBS, continuous theta burst stimulation; a/c-tDCS, anodal/cathodal tDCS; ES, electrical stimulation. Stimulation dosage/duration—d, day(s); m, month(s); w, week(s); y, year(s). Functional outcome measures—ALSFRS(-R), ALS functional rating scale (revised); MRC, muscle strength measured with the Medical Research Council scale; MVIC, maximum voluntary isometric contraction; FSS, fatigue severity scale; SF-36, MOS 36-Item Short Form Survey; ALSAQ-40, ALS assessment questionnaire; EQ-5D, EuroQol 5 dimensions; EQ-VAS, EuroQol visual analog scale; CBI, caregiver burden inventory; 6MWD, 6-min walking distance; 10MWT, 10-m walking test; TUG, timed up-and-go test; FSS, fatigue severity scale; BDI, Beck depression inventory*.

### Repetitive Transcranial Magnetic Stimulation

Most therapeutic trials employed rTMS with paradigms of stimulation known to have inhibitory effects on motor cortical excitability. In particular, cTBS was tested in four studies by Di Lazzaro et al. In a first randomized trial on 20 ALS patients, active bilateral cTBS of M1 delivered for 5 days/month was associated with slowing of disease progression after 6 months of treatment (88). This finding was not replicated in a following trial in which the duration of cTBS treatment was extended to 12 months (90). Of note, in a further small study by the same group (93) in which the dosage of cTBS was doubled to 10 days/month, a tendency to a slower progression over 6 months was observed when comparing with pooled data of patients treated for 5 days/month and of “sham”-treated patients from the previous studies (88, 90), even if it was not possible to demonstrate a reduction from baseline progression in this new patients' group (93). The other two studies with cTBS are small case series in which stimulation was delivered for a prolonged period of >2 years in one subject (91) or for a period of 1 year as an “open-label” extension study of previous trial in three subjects who had been observed for 1 year under placebo stimulation (92): in both cases, a reduction of ALSFRS-R score was observed in comparison with baseline observation. One study (101) confirmed, in a sample of 10 ALS subjects, the physiological inhibitory effect of cTBS on M1 excitability observed in the healthy population, by showing a cumulative increase of motor threshold and a reduction of MEP amplitude over 5 days of M1 stimulation, supporting the rationale for testing this kind of stimulation in ALS.

One earlier pilot study tested both low-frequency (1 Hz) rTMS in two subjects and high-frequency (20 Hz) rTMS in two subjects (87): the rationale for using high-frequency stimulation, with an excitatory effect on M1, was provided by another study that showed that 20-Hz rTMS can induce an increase of brain-derived neurotrophic factor (BDNF) blood level (102). However, only 1-Hz rTMS was associated with slowing of disease progression over more than 2 years of treatment, while 20-Hz rTMS did not determine any reduction in baseline progression, which instead had an acceleration in the two treated patients, and it was therefore discontinued within 3 months. The latter finding could be explained by the fact that high-frequency rTMS enhances the activation of non-NMDA glutamatergic receptors within the motor cortex (103) with a consequent potentiation of non-NMDA-mediated glutamatergic excitotoxicity (104).

Another pilot controlled trial tested 5-Hz rTMS, delivered to M1 bilaterally for 2 weeks, in five ALS subjects (89). The rationale behind the use of this frequency of stimulation was looking for a compromise between the supposed detrimental cortical excitatory effect and protective BDNF-release-facilitating effect of high-frequency rTMS (102). A transitory improvement of muscle strength and quality of life was observed in the treated group; however, this effect was no longer evident 2 weeks after discontinuation of rTMS.

### Non-invasive Electrical Stimulation

Non-invasive techniques using electrical stimulation encompass tDCS and transcutaneous spinal stimulation. At present, other techniques of brain stimulation using a variable current intensity, such as transcranial alternating current stimulation (tACS), have not been employed in therapeutic trials in ALS.

In the last decade, tDCS was tested mainly in the attempt of obtaining a portable therapeutic option that does not require to be delivered within the hospital setting. Indeed, tDCS requires only a small size stimulator and it can be delivered even at patients' home.

To date, the number and size of tDCS studies is overall even smaller than that of rTMS studies. In the only study (94) in which cathodal tDCS of M1 was delivered for a prolonged time in a single subject, no effect on disease progression was observed. This was also the case of a single patient reported by Madhavan et al. (96), treated for 4-week periods with either anodal, cathodal, or sham tDCS, and of two patients reported by Sivaramakrishnan et al. (97), treated for 8 weeks with anodal tDCS of M1. The latter study also evaluated the feasibility of remotely supervised treatment, with therapists guiding caregivers through internet in the application of tDCS and reporting of adverse events.

From a physiological point of view, Munneke et al. (105) had failed to induce a consistent decrease in corticospinal excitability in ALS patients, compared to healthy controls, following a single session of 1-mA cathodal tDCS over M1.

Recently, Benussi et al. (95) tested a more complex tDCS paradigm, consisting in concurrent bi-anodal motor cortex and cathodal spinal stimulation (i.e., corticospinal tDCS), in the attempt to obtain a synergic effect from the stimulation of both structures. Thirty patients were recruited using a randomized controlled trial design. A significant improvement/stabilization in clinical scores of muscle strength, in quality of life scores, and in caregiver burden was observed after 2-week treatment with real tDCS applied 20 min/day at an intensity of 2 mA. Interestingly, these effects persisted at 6 months after the end of treatment and were paralleled by restoration of TMS parameters of intracortical circuit excitability (i.e., SICI and ICF). However, it must be observed that a slower-than-usual disease progression, as expressed by a decline of ALSFRS-R score of <2 pts over 6 months, characterized both real and sham tDCS groups. A further clinical trial by the same group is ongoing, with the aim of assessing stabilization of improvement after repeated treatment (ClinicalTrials.gov ID: NCT04293484).

A recent feasibility neurophysiological study (106) also confirmed the possibility of activating spinal motor neurons both trans-synaptically and non-synaptically, at axonal level, with cervical transcutaneous constant-current stimulation, supporting the possibility of using non-invasive tools for targeting spinal pathophysiological mechanisms of ALS. A pilot study is currently active in ALS to expand the investigation on the interaction of cervical transcutaneous stimulation with other central and peripheral circuits and with circuits involved in volitional limb movement (ClinicalTrials.gov ID: NCT03411863).

### Invasive Stimulation Methods

Invasive cortical or spinal stimulation might represent an interesting translational prospect of preliminary findings obtained non-invasively into therapeutic strategies based on increased stimulation dosage. However, to date, only very few cases of ALS patients with implanted stimulators have been reported.

Cortical subdural stimulation was initially tested by Sidoti and Agrillo (98) in four patients who underwent bilateral M1 stimulation for up to 2 years: a slowing of ALS progression was observed in two of them, while one committed suicide. Based on this experience, Di Lazzaro et al. (99) treated one subject, showing prominent upper motor neuron involvement at disease onset and a rapidly progressive course, with bilateral epidural M1 stimulation delivered for more than 3 years: while he did not show substantial changes in disease progression in the first 2 years, as measured with the ALSFRS-R, he was still alive at 12 years and he neither required continuous ventilatory support nor gastrostomy for about 10 years. The benefit of epidural motor cortical stimulation was confirmed in a SOD1-G93A murine model of ALS (107).

The effects of epidural spinal stimulation in ALS have been investigated by Di Lazzaro et al. (100) in one subject affected with a typical spinal-onset form of ALS. This subject was observed clinically for 10 months after starting cervical epidural spinal stimulation for the treatment of chronic cervico-dorso-lumbar pain: while high-frequency stimulation was effective in controlling pain, indicating that spinal cord circuits were effectively activated and modulated, it did not modify ALS progression compared to that before intervention.

### Safety Considerations

The studies analyzed above, including a total of <100 ALS patients undergoing active treatments with either rTMS, tDCS, or invasive subdural/epidural stimulation, did not report any adverse event. Mild dysesthesias under tDCS electrodes might be perceived (97). It must be noticed that, since studies did not investigate systematically the occurrence of mild collateral effects, e.g., using specific questionnaires, some of the most common mild effects already described in the guidelines on the use of rTMS and tDCS (108, 109), such as headache, might have not come to researchers' attention. It must also be considered that people affected with ALS have a usually high expectation for some efficacy of the proposed treatment; therefore, they might not pay enough attention to very mild collateral symptoms.

Seizures are the most feared adverse event with non-invasive brain stimulation, although they are a rare event. No seizures have been reported in the above-analyzed studies with ALS patients. Inhibitory rTMS and tDCS protocols are traditionally considered at lower risk of inducing seizures. A recent analysis on more than 300,000 TMS sessions (110) indicates that, when TMS is delivered within published guidelines (108), seizures have no higher incidence with high-frequency rTMS protocols than with low-frequency and single-pulse protocols, being it very low (~0.02/1,000 TMS sessions); otherwise, the risk is increased in subjects with other risk factors (~0.33/1,000 TMS sessions). In ALS patients, the effort toward increasing stimulation dose to strengthen therapeutic effects could lead to exceeding current safety limits: this might determine an increased risk of seizures, especially with continuous delivering of cortical epidural stimulation. However, given the low absolute risk of seizures in subjects without other risk factors (110), it is foreseeable that, if a higher stimulation dosage is proven to determine a clinically significant effect, the therapeutic window will be large enough to allow a more intensive intervention. Pauses between stimulation sessions could also help to reduce the risk of seizures.

Finally, due to the relatively short time of observation and the small number of recruited patients in ALS trials, long-term effects of prolonged brain stimulation procedures could not be investigated.

## Measuring the Effects of Therapeutic Interventions

Measuring the effects of new therapeutic interventions in ALS is challenging. Besides survival, requiring large samples, and long trials, more selective indices of disease course are usually employed to increase sensitivity in small trials. However, distinct measures have a different sensitivity in catching specific functional and pathophysiological alterations that can be differentially affected during disease progression.

In this section, we summarize the main outcome measures that can be used to assess the response to neuroprotective interventions based on brain stimulation techniques, ranging from functional clinical scales measuring the overall level of disability to neurophysiological tools assessing specific dysfunctional mechanisms implicated in ALS pathophysiology (Table 2).

**Table 2:** Measures to evaluate the effects of therapeutic interventions.

	**Advantages**	**Disadvantages**
**Functional scales**		
**Overall clinical disability**		
ALSFRS-R	• Easy to employ, can be self-administered and used remotely for telehealth • Has been shown to correlate to survival and is robust in several trials	• Relatively insensitive to progression over short periods of time • Affected by mood/effort • Neglects clinical spectrum of UMN damage
King's system	• Easy to employ • Good estimation of disease progression, especially in early to mid-disease	• Ordinal scale • Low clinical resolution
MiToS staging systems	• Easy to employ • Good estimation of disease progression, especially in late stages	• Ordinal scale • Low clinical resolution
**Muscle strength**		
MRC	• Easy to use in clinical setting • Good overview of muscle wasting pattern	• Subjective, affected by the mood of both patient and examiner • Not sensitive to disease progression • Ordinal scale • Poor reproducibility • Not able to distinguish weakness due to LMN vs. UMN
Quantitative testing (dynamometer)	• Reliable and sensitive indicator of disease progression • Good reproducibility • Objective measure	• Usually limited to hand muscles • Not able to distinguish weakness due to LMN vs. UMN
**Respiratory function**		
FVC	• Quantifies the health of the most critical activity for sustaining life • Repeatable	• Only evaluates a single region • May not show changes early in disease course • Later in disease, it may be affected by bulbar weakness and poor lip closure
**Cognition**		
ECAS, ALS-CBS, BBI	• Validated screening instruments • Objective measures of cognitive decline	• Language and fluency scores can be partly biased by reduced fine motor skills and/or speech impairments • Require specialized equipment and training
**Neurophysiological measures**		
**UMN dysfunction**		
TMS: CMCT	• Easy to use in clinical setting • Can be used in multicenter studies	• Affected by muscle wasting • Not accurate for early UMN dysfunction • Not sensitive enough for proximal muscles
TMS: threshold tracking method	• Good biomarker in the assessment of UMN dysfunction • Monitor the effects of new drugs on UMN	• Requires specialized equipment and training • Few normative data available • Limited to distal muscles • Not able to capture proximal muscle involvement • May be challenging to use in multicenter studies
TMS: TST	• Allows a good estimation of the proportion of lost UMNs supplying the target muscle • TST reductions are more common than abnormalities of conventional MEP (i.e., prolonged CMCT or reduced MEP amplitude)	• Quite painful • Few normative data available • Cannot be performed to proximal muscles • Requires specialized equipment and training • May be challenging to use in multicenter studies
**LMN dysfunction**		
CMAP	• Easy to obtain • Can be used in multicenter studies	• Limited to distal muscles • Not able to capture proximal muscle involvement • Insensitive in assessing LMN degeneration due to reinnervation
NI	• Easy to obtain, requires no special equipment • May be very sensitive to LMN loss, even in pre-symptomatic stage • Sensitive indicator of disease progression	• Limited to distal muscles • Not able to capture proximal muscle involvement • Requires persistent F-waves • Few normative data are available
MUNE/MUNIX	• Sensitive to LMN loss, even with stable CMAP amplitudes • Provides approximation of actual motor neuron number and rate of actual motor neuron loss	• Technically challenging • Limited to distal muscles • Not able to capture proximal muscle involvement • Low repeatability especially at the early stage of disease
F-wave	• Indirect measure of spasticity • Good measure of LMN excitability	• Limited to distal muscles • Not able to capture proximal muscle involvement • Correlates poorly with clinical deficit • Usually requires an adequate CMAP amplitude
Peripheral axonal excitability testing	• Provides information about peripheral axonal excitability • Potentially useful for early diagnosis • Good biomarker of therapeutic effectiveness • Correlates with survival	• Limited data on progression • Requires specialized equipment and training • May be challenging to use in multicenter studies • Varies a lot among axons in a single nerve • Limited to upper limbs

### Functional Scales

#### Overall Clinical Disability

Initial attempts for developing a functional rating scale were pursued in the 1970s, namely, the Norris Scale and its modified version that consists of two parts, i.e., the Limb Norris Score, composed of 21 items, and the Norris Bulbar Score, composed of 13 items (111). However, both scales were complex and time consuming to administer. Thus, in the 1990s, the most well-known and utilized measure of disease progression, the ALS functional rating scale (ALSFRS), was developed (112). Few years later, the original ALSFRS was replaced with the ALSFRS-revised (ALSFRS-R) when it was recognized that additional measures of respiratory decline were needed (113). The new scale consists of 12 items (highest score: 48) encompassing three main domains: bulbar function, fine and gross motor abilities, and respiration. An extended version of the ALSFRS-R (ALSFRS-EX) has also been proposed to improve its sensitivity at lower levels of physical function in patients in an advanced stage, by adding three items to ALSFRS-R relating to the ability to use fingers, to show emotional expression in the face, and to get around inside the home (114).

ALSFRS-R is largely used in several clinical trials, it is easy to perform remotely for telehealth, and its self-administered version shows good reliability and sensitivity to change over time vs. the standard evaluator-administered ALSFRS-R (115). However, this scale has several limitations that should be acknowledged (Table 2). A recent Rasch analysis found that the test failed rigorous measurement standards, with the authors of this analysis calling for its revision (116). Lastly, the ALSFRS-R score largely reflects disability due to muscle wasting, mostly representing the LMN involvement; therefore, it neglects clinical spectrum of UMN damage. To obtain a more specific scale of UMN involvement, patients can be also graded in terms of UMN “burden,” by totaling the number of pathological UMN signs on examination, such as brisk upper and lower limb reflexes and brisk facial and jaw jerks (highest score: 16) (117). Additional scores for UMN degeneration are the Penn UMN Score (118, 119), measuring also pseudobulbar symptoms by considering the Center for Neurologic Study-Lability Scale (CNS-LS) (120), and muscle tone by means of the Modified Ashworth scale (MAS) (121).

Importantly, over the last few years, two functional scales [the King's College and Milano-Torino Staging (MiToS) staging systems] have been proposed to staging ALS. The King's College system uses five stages, ranging from 1 being symptom onset and stage 5 being death, and relies on the clinical spread of disease among the several clinical regions as a measure of progression (122). Instead, the Milano-Torino Staging (MiToS) (123) utilizes the subscores produced by the ALSFRS-R to define stage, with stage 0 being normal function and stage 5 being death. A recent comparison analysis suggested that the two systems are complimentary, with King's staging showing greatest resolution in early to mid disease and MiToS staging having higher resolution for late disease (124).

#### Muscle Strength

Loss of muscle strength is a cardinal feature of ALS. Functional loss over time, including respiratory dysfunction, inability to ambulate, loss of ability to perform activities of daily living, and others are due, in large part, to decline in strength. Therefore, the accurate measurement of limb muscle strength and respiratory function is essential in therapeutic trials to better understand the impact of therapy on vital function.

With regards to limb muscle strength evaluation, we have a qualitative assessment with the manual muscle testing (MMT) scale established by the Medical Research Council of the Royal College of Physicians and Surgeons (MRC), scoring from 0 (paralysis) to 5 (normal strength) each tested muscle. However, the measure itself is subjective; therefore, to overcome such limitations, quantitative scales have been developed (i.e., muscle strength evaluation with dynamometer), and with careful training and reliability testing, they have proven to be reliable and sensitive indicators of disease progression. The ability to determine objectively the source of weakness would be of great value and should be a subject of future research (125).

#### Respiratory Function

Respiratory failure is the primary cause of death in ALS. Interestingly, approximately in 3–5% of cases, ALS can begin with respiratory failure (126–128). Change in respiratory performance is indicative of ALS progression, since respiratory function is directly related to skeletal muscle function and survival (129).

According to American (130) and European guidelines (131), all ALS patients should perform regularly after diagnosis spirometry measurements, such as forced vital capacity (FVC). Other recommendations include nocturnal pulse oximetry, polysomnography, arterial blood gases, maximal inspiratory/expiratory pressure, sniff nasal pressure, or trans-diaphragmatic pressure if patients are symptomatic and FVC is >50% (132). The inclusion of these tests, in addition to FVC, may assist in detecting changes in respiratory function early in the disease course (133) and leading to institution of supportive therapy with non-invasive ventilation (130, 131).

#### Cognition

Although motor system deficits may appear prominent, ALS is increasingly recognized as a multisystem disorder accompanied by cognitive changes (128).

In contrast to the relentlessly progressive motor deficits, the trajectory of cognitive and behavioral deficits is less clear due to considerable individual variations, genotype-associated profiles (134, 135), differences in assessment strategies, and practice effects (136). Importantly, recent evidence has pointed out that up to 30–50% of ALS patients show cognitive impairment, ranging from frontotemporal dementia to milder forms of executive or behavioral dysfunctions (128). The impairment of cognitive functions is a relevant negative prognostic risk factor, independent from other known factors such as age, site of onset, diagnostic delay, disease severity, and respiratory function (137, 138). In addition, cognitive impairment can be present at diagnosis as well as manifest during the disease (139), and it might get worse in parallel with motor deterioration (140), and patients with respiratory compromise are more likely to develop reduced cognitive function (141).

Cognitive and behavioral domains are routinely assessed thanks to validated screening instruments such as the Edinburgh Cognitive and Behavioral ALS Screen (ECAS) (142), the Beaumont Behavioral Inventory (BBI) (143), and the ALS Cognitive Behavioral Screen (ALS-CBS) (144).

### Survival and Combined Assessments

Since ALS is characterized by progression to death, survival is recommended as the primary endpoint for phase 3 trials (145). Indeed, the approval of the first drug for ALS, riluzole, was based on survival data (4).

Survival analysis, in its wider conception, encompasses approaches to investigate the time for an event of interest to occur and is largely based on Kaplan–Meier estimates. Since the time to death in ALS is extended by nutritional and respiratory interventions, some trials included time to tracheostomy or prolonged non-invasive ventilation as part of survival outcome, but the use of respiratory interventions differs between centers and it could even increase variability of these combined measures (145).

Considering that survival time in ALS is quite variable, in order to reach a sufficient statistical power, the two fundamental requirements are that (a) the time of observation must be long enough relative to the average disease course and (b) the groups must be large enough to overcome intrinsic variability and appreciate differences. Survival analysis is also biased by censored patients, i.e., those subjects who are missed at follow-up for any reason and exit the population “at risk,” thus flattening the end of the survival curve. If the number of censored subjects is high, and this might well be the case in long-lasting ALS trials, it will affect the accuracy of survival estimation. Moreover, the above requirements are not satisfied by pilot studies, which usually aim to explore the possibility of a further investment on a new treatment strategy and therefore switch to functional or physiological outcome measures.

Traditionally, survival and function have been assessed as independent endpoints in ALS trials. Combined approaches have been implemented to reduce the confounding effect of mortality on analysis of functional outcomes. Most notably, the Combined Assessment of Function and Survival (CAFS) calculates a rank for each subject, based on survival time and decline in the ALSFRS-R score: in this way, a mean rank score can be calculated that allows for statistical comparison between treatment groups (146). Additional analytical strategies, such as joint models, have been implemented to overcome the limitation of CAFS in detecting functional effects when the rate of mortality is high (147).

### Neurophysiological Measures

Neurophysiological techniques might provide an objective measure of UMN and LMN dysfunction in ALS. For this reason, they seem to be more sensitive to detect disease progression with respect to clinical scales. Neurophysiological abnormalities might also uncover UMN and LMN deficit that are clinically silent, thus enabling an earlier diagnosis and thereby recruitment into therapeutic trials. Additionally, they could provide important insights into disease mechanisms that ultimately lead to uncovering of novel therapeutic targets.

#### Biomarkers of UMN Dysfunction

Single and paired-pulse TMS techniques, providing parameters such as MT, central motor conduction time (CMCT), cortical silent period, and intracortical inhibition and facilitation, have gained credibility as a clinical tool to investigate the integrity and excitability of the corticomotoneuronal system (see *Neurophysiological Insights* section above).

Reduced SICI, above all, was reported to be an independent prognostic biomarker in ALS patients within the first 2 years of disease onset (148), and in a separate study, SICI was shown to be partially normalized with riluzole treatment (149). Paralleling the clinical efficacy of riluzole, the modulating effects lasted about 2 months (150).

Regardless of the underlying mechanisms, studies of riluzole have suggested a utility of TMS in assessing biological effectiveness of compounds at an early stage of drug development. These results suggest that non-invasive *in vivo* monitoring of cortical function, and particularly SICI, may also be an effective biomarker used to monitor the effects of novel therapeutics in a clinical trial setting.

A limitation of the conventional paired-pulse technique has been the marked variability in MEP amplitudes with consecutive stimuli. One of the solutions against this variability problem is the threshold tracking method, which was developed for paired-pulse TMS studies and suggested to be a good biomarker in the assessment of UMN dysfunction in ALS (17, 151). Another method to overcome this difficulty is the triple stimulation technique (TST) that provides a measure for conduction failure (152).

As discussed in a section above, it is still not defined how TMS metrics change over time. Thus, future studies are needed to explore the reasons of heterogeneity in cortical excitability changes during ALS progression and to identify possible clinical phenotypes and disease trajectories associated to cortical inexcitability or hyperexcitability.

Even if promising, TMS techniques have some limitations for disease monitoring (Table 2). Current neurophysiological methods do not explore function in most of the ancillary UMN pathways (i.e., tectospinal, rubrospinal, vestibulospinal, and reticulospinal tracts, as well as various short internuncials and cerebellar connections), which have a critical role in the disease process. In addition, the role of UMN pathways within the spinal cord, an integral component of the central nervous system, is not well-defined.

#### Biomarkers of LMN Dysfunction

Conventional neurophysiological techniques, such as nerve conduction studies which measure the compound muscle action potential (CMAP) amplitude, may be relatively insensitive in assessing LMN degeneration due to the process of reinnervation (153).

The neurophysiological index (NI), using a simple formula, combines routine CMAP amplitude, F-wave frequency, and distal motor latency to improve sensitivity in demonstrating longitudinal LMN loss in ALS (154). NI is also able to detect LMN loss in muscles of the pre-symptomatic limb as well as successfully tracking disease progression, demonstrating continued loss of functional motor units during this pre-symptomatic period, when weakness, atrophy, or fasciculation are not detectable to both patients and clinicians (155). The validation of NI was also demonstrated longitudinally in the symptomatic muscles and correlated with ALSFRS-R decline (156, 157). Additionally, NI is able to detect deterioration that occurred over a short period of 4 weeks in ALS patients, hence enabling the utility of this index to monitor treatment efficacy (158). Therefore, NI is sensitive to LMN loss and weakness in ALS, whether the disease is rapidly or slowly progressive.

Regarding the use of F-waves, cortical and peripheral mechanisms have been proposed to account for the F-wave abnormalities (159, 160). F-wave frequency together with H-reflex amplitudes and recovery curves are considered as an indirect measure of spasticity (159), but they correlate poorly with clinical deficit (161, 162).

Motor unit number estimations techniques, such as motor unit number estimation (MUNE) and motor unit number index (MUNIX), may potentially represent valuable biomarkers of LMN degeneration. Studies utilizing various MUNE techniques in ALS patients and healthy controls have reported good intra-rater and inter-rater reliability (163–165). In addition, progressive linear decline in MUNE counts has been reported in ALS (166), suggesting utility as a potential biomarker of disease progression in a clinical trial setting (164). The MUNIX technique is a method designed to express the number of functioning motor units within a muscle as an index, instead of providing a direct measure of their absolute numbers. Recent studies using different MUNE methods have demonstrated potential utility for assessing disease progression in ALS patients as reflected by a progressive linear decline in MUNE counts (164, 166–169). Interestingly, a recently developed MUNE technique, termed MScan, appeared to be the most sensitive MUNE method in detecting ALS disease progression (169). Additionally, MUNIX was able to detect disease progression in pre-symptomatic muscles in ALS (170, 171), and longitudinal changes in these muscle groups appeared more sensitive than ALSFRS-R (168).

Peripheral axonal excitability testing provides information about nodal and internodal axonal ion channel function. Importantly, the changes in axonal excitability have been linked to development of muscle cramps, fasciculation, and motor neuron degeneration (172–174) and have been associated with a shorter survival (175, 176). Of relevance, a cross-sectional and a subsequent longitudinal study demonstrated the effect of riluzole in inducing a significant reduction of both axonal refractoriness and superexcitability (17), and these peripheral modulating effects lasted at least 2 months (150). In addition, excitability measures have also been utilized in clinical trials assessing the effects of Na^+^ channel-blocking agents. Specifically, low-dose mexiletine (300 mg) did not exert any modulating effects on axonal excitability parameters, potentially accounting for the absence of clinical effectiveness (177). Separately, axonal excitability studies disclosed stabilization of axonal ion channel function in patients with ALS treated with the Na^+^ channel-blocking agent flecainide (178), associated with a reduced rate of LMN dysfunction. These clinical studies underscore the potential of utilizing axonal excitability parameters as biomarkers of therapeutic effectiveness in a drug trial setting. Of note, a recent phase 2 trial assessing efficacy of retigabine, a potassium channel activator, used successfully axonal excitability parameters as secondary outcome measures (179).

## Discussion and Perspective

Findings of studies on the clinical effects of NIBS in ALS patients suggest a possible efficacy in determining a slight reduction of disease progression, related to the duration and frequency of treatment. However, results were quite variable and of limited clinical significance. Previous systematic reviews on rTMS for the treatment of ALS resulted in insufficient evidence on the efficacy of this type of intervention (180, 181). Latest studies with rTMS and new methods of stimulation did not add data that could substantially modify the picture represented by previous analyses in terms of clinical efficacy; thus, the current evidence must be considered as preliminary.

Several reasons related to experimental design can account for the lack of clinically consistent effects. One main limitation was the lack of a sufficient power to detect small size effects on selected outcome measures, which did not allow to obtain informative results in the case of both positive and negative outcomes. Indeed, all studies in ALS until now, while attempting to test therapeutic efficacy, recruited no more than 20 subjects in the active treatment group and did not provide any information on dose–response relationship, in terms of both tolerability and efficacy, and therefore do not go beyond the stage of phase 1 trials (safety evaluation). Moreover, an intrinsic limitation in this field relates to the fact that most rTMS studies were conducted by the same research group.

Related to the previous point, it is a matter of great importance and complexity selecting suitable endpoints, to which we have dedicated a section of the present work. Indeed, distinct outcome measures have a different sensitivity in catching specific functional and pathophysiological alterations that can be differentially affected during the disease course. Trials with prolonged (several weeks or months) interventions substantially used functional and strength scores as the main outcome measures, with the ALSFRS-R almost constantly employed. These outcome measures are subject to high variability among individuals and along disease progression. The existence of even infrequent “plateaux” and “reversals” in the ALSFRS score decline (182) further highlights the importance of an appropriate sample size and limits the reliability of conclusions drawn from individual case reports. It is quite natural to reason that, in a disease characterized by rapid progression to death, an effective treatment should increase the survival rate (or the time interval to respiratory failure). However, none of the reviewed studies has a long enough time of observation and a large enough cohort to allow for a sufficient power in evaluating survival. In fact, survival was considered as a main efficacy outcome only in reports of individual cases deviating from the usual disease course [e.g., (91, 99)]. Nonetheless, in possibly subtherapeutic and/or undersized trials, functional and neurophysiological endpoints remain useful for preliminarily evaluating the potential biological effects of a given treatment protocol and for obtaining information on the feasibility of larger studies. In this context, using objective neurophysiological markers of UMN and LMN dysfunction could help to detect the biological effects of interventions targeted to specific pathophysiological mechanisms in a preliminary phase; before that, frequency of administration and dosage are eventually optimized to obtain therapeutic effects, while it has been questioned that measures of central motor function can be useful for monitoring patients in a clinical trial setting (26). It should also be considered that neurophysiological monitoring increases the load of experimental procedures and limits the number of patients who can be recruited.

A second important limitation in ALS trials is the heterogeneity of recruited patients. Inclusion of subjects with different phenotypes and/or disease stages considerably increases the within-group variability and required sample size. Recruitment difficulties, mainly related to the high effort required for patients to regularly reach clinical centers to undergo NIBS procedures and the difficulty to plan the exclusion of many potentially highly motivated subjects in the absence of other therapeutic options, could account for study protocols with wider inclusion criteria.

The disease severity and duration at the time of enrolment also plays an important role in the evaluation of treatment outcome, for two main reasons. First, ALS progression, as evaluated by the ALSFRS score, typically slows in the more advanced stages (183) and this can mask treatment effects. Second, based on the hypothesis that hyperexcitability primes neurodegeneration, experimental treatments aimed at counteracting hyperexcitability might not be effective in an advanced stage or in rapidly progressive disease forms, where neuropathological alterations leading to degeneration have already occurred. It should also be considered that cortical and spinal structural changes that might accompany advanced degeneration could alter the effects of the applied electric field; however, a clear cortical atrophy is demonstrated only in ALS subjects with a frontotemporal degeneration phenotype (184, 185).

Dealing with the purpose of early recruitment is challenging since the sensitivity of the clinically based criteria is limited, particularly in the early stages of ALS (186–188), leading to significant diagnostic delays and thereby a delay in institution of neuroprotective therapies and recruitment into therapeutic trials (189). To this end, neurophysiological techniques might be more sensitive in detecting UMN and LMN deficits that are clinically silent, thus enabling an earlier diagnosis and disease-modifying interventions. For example, it was suggested that biomarkers that can detect changes in the integrity of the corticomotoneuronal synapse should be able to identify the earliest stages of ALS (190).

Besides limitations related to experimental design, the characteristics and neurobiological effects of brain stimulation interventions must be considered. We described how different NIBS protocols interact with corticospinal circuitry. Protocols targeting indirect intracortical connections with CSNs (i.e., those affecting later I waves), such as 1-Hz rTMS, PAS, and cathodal tDCS, have the potential to interact with mechanisms of hyperexcitability related to hypoactive GABAergic interneurons, demonstrated by neurophysiological and neuropathological investigations. Otherwise, protocols targeting direct projections to CSNs or CSNs themselves, such as cTBS and possibly tDCS, could reduce intrinsic UMN excitability. However, based on current knowledge, it is not possible to foresee which of the above hypothetical mechanisms (upstream or direct interaction with CSNs) will be more efficient. This is substantially due to a still insufficient knowledge on the pathological cascade at single-cell level and on the exact interaction of brain stimulation techniques with individual cell populations. Moreover, we do not know to which extent counteracting corticospinal hyperactivity might prevent LMN degeneration (191), even if this approach has a strong rationale in the theory of cortical-driven excitotoxicity.

Studies on ALS patients also do not provide sufficient indications on what might be the best stimulation paradigm. To date, cTBS was tested in the majority of patients, with some favorable results in the first months of treatment (88) and at higher dosage (93). The 1-Hz rTMS showed promising results but it was tested only in a very preliminary study (87). Cathodal tDCS seems to be ineffective in preliminary observations (94, 96, 105), while a recent wider study suggests a functional effect of corticospinal tDCS (95) but limited by the fact that stimulation was applied only for 2 weeks (Table 1). Of note, with tDCS, individualized electric field modeling (192, 193) could improve stimulation targeting and dosing of current density in target brain areas.

Experimental data also suggest that hyperexcitability in ALS might be a compensatory phenomenon of cell loss (191): in this case, inhibitory brain stimulation protocols would not be effective in slowing progression of pathogenic processes. However, facilitatory rTMS protocols, which might ease this compensatory phenomenon, seem ineffective, even if tested in very preliminary studies (Table 1).

Finally, it must be accepted that, if most or the totality of ALS forms depend on gene alterations and if hyperexcitability is not a causal factor of neuropathological alterations and cell loss, all interventions targeted to counteracting hyperexcitability can be expected, at best, to slow the disease progression without substantial effects on long-term survival.

In any case, since the physiological effect of NIBS techniques on cortical excitability is usually short-lasting, the time during which the motor cortex is exposed to stimulation appears as a key factor to produce clinically significant effects. The application of rTMS protocols is limited by the necessity of being administered within the hospital, while tDCS could be implemented for home self-administration. The administration at the patient's site of a new NIBS technique, transcranial static magnetic field stimulation (194, 195), has been recently proposed in ALS (196) and is currently being tested in a randomized controlled trial (ClinicalTrials.gov ID: NCT04393467). Alternatively, implanted devices could warrant prolonged daily stimulation, but their interaction with intracortical circuits is still largely unexplored.

In conclusion, in our view, there are few approaches that could warrant informative results from future trials.

- Targeted patients' selection. Subjects in the early stages of disease, stratified based on clinical characteristics and biomarkers, could represent a population exhibiting less variability and more likely to benefit of the potential disease-modifying effect of brain stimulation interventions.
- Adequate sample size. Samples larger than those tested until now, which could be obtained with multicenter studies, will allow to perform phase 2 or phase 3 clinical trials once a promising intervention has been defined from pilot studies.
- Targeted stimulation protocols with appropriate outcome measures. Further research on the mechanisms of corticospinal dysfunction in ALS and on the way to target specific cortical or corticospinal circuits will allow to target specific pathophysiological mechanisms. Neurophysiological or molecular biomarkers could allow to detect biological effects of proposed treatments before their clinical translation.
- Ensuring suitable stimulation dosage. Based on the current knowledge on the time course of neurophysiological effects of most brain stimulation techniques, it appears that protocols tested until now are likely underdosed to produce clinically significant effects. Increased dosage can be ensured with either repeated sessions of self-administered non-invasive stimulation or continuous stimulation delivered through implanted devices. The best risk-to-benefit ratio for different protocols remains to be determined.

## Author Contributions

All authors listed have made a substantial, direct and intellectual contribution to the work, and approved it for publication.

## Conflict of Interest

The authors declare that the research was conducted in the absence of any commercial or financial relationships that could be construed as a potential conflict of interest. The handling editor declared a past co-authorship with one of the authors VD.
